# The Roles of Interleukin-18 in Inflammation and Autoimmune Disease

**DOI:** 10.1007/s12016-026-09139-5

**Published:** 2026-03-16

**Authors:** Yue Zhao, Yuan Tang, Xiaoxia Zhu, Yunxia Hou, Hongbin Li, Francesco Ciccia, Hejian Zou, Liwei Lu

**Affiliations:** 1 Department of Pathology and Shenzhen Institute of Research and Innovation, The University of Hong Kong, Hong Kong, China; 2 Centre for Oncology and Immunology, Hong Kong Science Park, Hong Kong, China; 3 Department of Rheumatology, Huashan Hospital, Fudan University, Shanghai, China; 4 Department of Rheumatology and Immunology, Inner Mongolia Key Laboratory of Pathogenesis and Immunodiagnosis of Rheumatic and Immunological Diseases, The Affiliated Hospital of Inner Mongolia Medical University, Hohhot, China; 5 Rheumatology Unit, Department of Precision Medicine, Università degli Studi della Campania “L. Vanvitelli”, Naples, Italy

**Keywords:** Interleukin-18, Interleukin-18 binding protein, Innate and adaptive immunity, Inflammation, Autoimmunity

## Abstract

Interleukin-18 (IL-18) is a member of the IL-1 cytokine family with significant homology to IL-1β. New emerging evidence indicates that IL-18 acts as a pivotal molecular bridge connecting innate and adaptive immunity, functioning through tightly regulated mechanisms involving inflammasome-dependent and -independent proteolytic processing. The activity of IL-18 is primarily maintained by IL-18 binding protein (IL-18BP), which neutralizes IL-18 under physiological conditions. However, dysregulations in the IL-18/IL-18BP axis result in excessive IL-18 bioactivity, driving tissue inflammation and damage closely associated with autoimmune pathogenesis. Herein, we review recent advances in IL-18 biology and its expanding roles in the development of various autoimmune diseases. The molecular mechanisms by which IL-18 mediates organ-specific pathology are discussed to highlight its diverse effector functions across different autoimmune conditions. In addition, we evaluate emerging therapeutic approaches targeting the IL-18 pathway, with a focus on their potential and challenges in translational clinical research.

## Introduction

Autoimmune diseases arise from dysregulations of both innate and adaptive immune responses, resulting in chronic inflammation and progressive tissue damage in multiple organs. These conditions are characterized by loss of self-tolerance, excessive activation of autoreactive T and B cells, and sustained overproduction of proinflammatory cytokines [1]. A key clinical challenge in managing autoimmune diseases is the limited performance of existing biomarkers for early detection, risk stratification, and accurate monitoring of treatment response [2]. Consequently, there is an urgent need to identify novel mediators, particularly those operating at the intersection of innate and adaptive immune dysregulation and cytokine-driven inflammation, that could enhance early diagnosis, therapeutic efficacy and ultimately improved long-term patient outcomes.

Interleukin-18 (IL-18), a pleiotropic member of the IL-1 cytokine superfamily [3], has emerged as a critical mediator bridging innate and adaptive immunity, with a growing body of evidence highlighting its pathogenic role in autoimmune diseases [4, 5]. IL-18 is predominantly synthesized as an inactive precursor by monocytes, macrophages, dendritic cells, Kupffer cells, keratinocytes, and epithelial cells, which is processed into its mature bioactive form primarily by inflammasome-activated caspase-1 [6]. Mature IL-18 exerts potent pro-inflammatory effects by binding to the IL-18 receptor α/β heterodimer expressed on T cells, natural killer (NK) cells, and myeloid cells, which triggers downstream signaling pathways including recruitment of myeloid differentiation primary response 88 (MyD88), interleukin-1 receptor-associated kinase 4 (IRAK4), and tumor necrosis factor receptor-associated factor 6 (TRAF6), thereby activating nuclear factor kappa-B (NF-κB), mitogen-activated protein kinase (MAPK), and activator protein-1 (AP-1) [7, 8]. Through these signaling cascades, IL-18 potently drives the differentiation of T helper 1 (Th1) and T helper 17 (Th17) cells, synergistically amplifying IFN-γ production while also enhancing the secretion of other key proinflammatory cytokines such as TNF-α, IL-1β, GM-CSF, and various chemokines. These downstream effects promote robust neutrophil and macrophage recruitment, upregulate cell adhesion molecules and augment Fas ligand-mediated cytotoxicity. Collectively, these mechanisms perpetuate chronic inflammation and drive sustained tissue damage [9].

In line with its function as a potent amplifier of inflammation, IL-18 is markedly elevated in inflamed tissues and circulation across various autoimmune diseases, including rheumatoid arthritis (RA), systemic lupus erythematosus (SLE), Sjögren’s syndrome (SS), inflammatory bowel disease (IBD) and adult-onset Still’s disease (AOSD) [3, 4]. Recent evidence further underscores its causal involvement, with elevated circulating IL-18 levels consistently associated with disease activity and progression, particularly in conditions involving inflammasome dysregulation such as AOSD and macrophage activation syndrome (MAS) [10]. Notably, circulating bioactive free IL-18 is strictly regulated by the high-affinity IL-18 binding protein (IL-18BP), a physiological inhibitor that sequesters IL-18 and prevents its systemic proinflammatory activity [11]. Disruptions in this IL-18/IL-18BP balance have been implicated in driving hyperinflammation and tissue damage. This review focuses on the pathogenic contributions of IL-18 and the disrupted IL-18/IL-18BP balance in autoimmune diseases, with specific emphasis on their emerging value as biomarkers for diagnosis, assessment of disease activity and promising therapeutic targets. The rapidly advancing understanding of IL-18 biology, evidenced by ongoing clinical trials of IL-18 inhibitors such as tadekinig alfa (recombinant IL-18BP), creates an urgent imperative to translate this knowledge into effective clinical tools, enabling targeted therapies and better patient outcomes.

## Biological Roles of IL-18

### Synthesis and Maturation

Originally identified as interferon-γ-inducing factor (IGIF) in 1989 in the context of *Mycobacterium bovis* BCG-infected and LPS-challenged mice [12], IL-18 was molecularly cloned in 1995 [13] and subsequently recognized as a member of the IL-1 cytokine superfamily based on its sequence similarity to IL-1β, conserved β-trefoil fold, shared Toll/interleukin-1 receptor (TIR)-domain signaling, and caspase-1-dependent maturation [13, 14]. Similar to IL-1β, IL-18 is synthesized as a biologically inactive 24-kDa precursor (pro-IL-18) that lacks a classical N-terminal signal peptide, preventing its entry into the conventional endoplasmic reticulum (ER)-to-Golgi secretory pathway. Pro-IL-18 can be either constitutively expressed or induced at high levels among monocytes/macrophages, dendritic cells, Kupffer cells, microglia, keratinocytes, intestinal and airway epithelial cells, and numerous non-hematopoietic stromal cells [15].

**Fig. 1:**
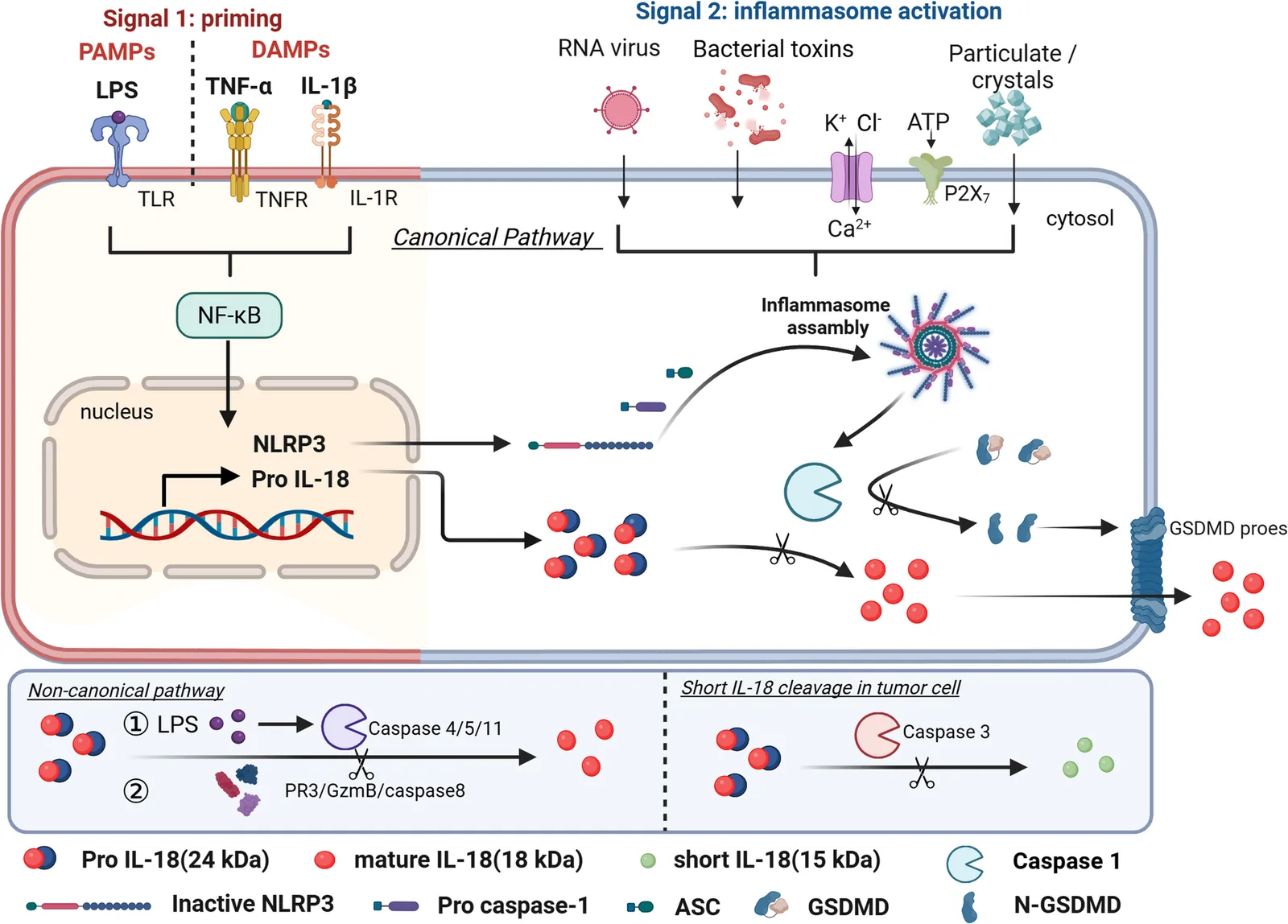
Mechanisms of IL-18 production and activation. In canonical pathway, IL-18 activation occurs through two sequential signals. Signal 1 (priming) is triggered by PAMPs such as LPS and DAMPs such as TNF-α or IL-1β, which lead to NF-κB-mediated transcriptional induction of NLRP3 and Pro-IL-18. Signal 2 (inflammasome activation) is stimulated by secondary triggers, including RNA viruses, bacterial toxins, ATP, or particulate matter that perturb ion fluxes (e.g., K⁺, Cl⁻, and Ca²⁺). These stimuli induce the assembly of the NLRP3 inflammasome complex, composed of NLRP3, ASC, and pro-caspase-1. Activated caspase-1 cleaves Pro-IL-18 (24 kDa) into its biologically active mature IL-18 (18 kDa) form. In parallel, caspase-1 cleaves GSDMD to produce N-terminal pore-forming domain, which forms membrane pores facilitating the secretion of mature IL-18. In the non-canonical inflammasome pathway, cytosolic LPS directly activates human caspases-4/5 (or mouse caspase-11), thus processing pro-IL-18 into bioactive mature IL-18. Proteases such as PR3, GzmB, and caspase-8 mediate separate caspase-1-independent pathways for pro-IL-18 cleavage. In certain tumor cells, Pro-IL-18 undergoes alternative cleavage by caspase-3, producing a truncated isoform named short IL-18 (15 kDa). This cytoplasmic form may possess distinct biological functions within the tumor microenvironment. PAMPs, pathogen-associated molecular patterns; DAMPs, damage-associated molecular patterns; ASC, Apoptosis-associated speck-like protein containing a caspase recruitment domain (CARD); PR3, Proteinase 3; GzmB, granzyme B

For many years, the maturation and release of bioactive 18-kDa IL-18 were thought to depend on caspase-1-mediated proteolytic cleavage of pro-IL-18 within canonical inflammasomes, including NLR family pyrin domain containing 3 (NLRP3), Absent In Melanoma 2 (AIM2), NLR family CARD domain-containing protein 4 (NLRC4), and Pyrin [16]. Over the past decade, however, several caspase-1-independent mechanisms of pro-IL-18 processing and secretion have been identified. Enzymes such as proteinase 3 (PR3) and granzyme B can directly cleave pro-IL-18 to generate bioactive IL-18 in tissues enriched with these proteases, such as psoriatic skin, rheumatoid synovium, and atherosclerotic plaques [17, 18]. Additionally, pro-IL-18 released from dying cells can be processed extracellularly by PR3 secreted from activated neutrophils, yielding bioactive IL-18 in the plasma of patients with systemic inflammatory diseases [19]. In certain epithelial cells and macrophages, engagement of Fas by FasL assembles a death-inducing signaling complex that recruits caspase-8, which can either indirectly activate caspase-1 or directly cleave pro-IL-18, thereby enabling rapid production of mature IL-18 in response to cytotoxic stress or viral infection [20]. Additionally, during pyroptosis or sublytic gasdermin D (GSDMD) pore formation, both mature IL-18 and pro-IL-18 are secreted directly through GSDMD pores, entirely circumventing the classical ER-Golgi secretory pathway [21]. Notably, caspase-3 functions as an inflammasome-independent protease that processes pro-IL-18 into a highly potent 15-kDa isoform capable of enhancing anti-tumor immunity [22]. These alternative pathways account for the sustained elevation of mature IL-18 observed in caspase-1- or NLRP3-deficient mice and in patients with cryopyrin-associated periodic syndromes (CAPS) treated with caspase-1 inhibitors, underscoring the potential value of therapeutic targets beyond the canonical inflammasome pathway to regulate dysregulated IL-18 in autoimmune and inflammatory diseases (Fig. 1).

### Signaling Pathways

IL-18 signaling is mediated by a heterodimeric receptor complex comprising the ligand-binding IL-18 receptor alpha chain (IL-18Rα, also known as IL-18R1) and the signal-transducing IL-18 receptor beta chain (IL-18Rβ, also known as IL-18RAP). The signaling cascade begins when mature IL-18 first binds with moderate affinity to IL-18Rα expressed on the surface of responsive cells, mainly T cells, NK cells, and certain myeloid subsets. This interaction induces a conformational change that facilitates the recruitment of IL-18Rβ, forming a high-affinity ternary complex that initiates intracellular signals [23]. Complex assembly triggers the canonical MyD88-dependent pathway. First, the cytoplasmic TIR domains of the receptor chains recruit the adaptor MyD88, which then engages IRAK family kinases (including IRAK4 and IRAK1/2). This scaffolding leads to TRAF6 activation, which in turn stimulates two major downstream cascades: the NF-κB pathway and the MAPK pathways, including extracellular signal-regulated kinases (ERK)1/2, p38, and c-Jun N-terminal kinase (JNK), Ultimately driving the transcriptional upregulation of proinflammatory genes [24, 25].

**Fig. 2:**
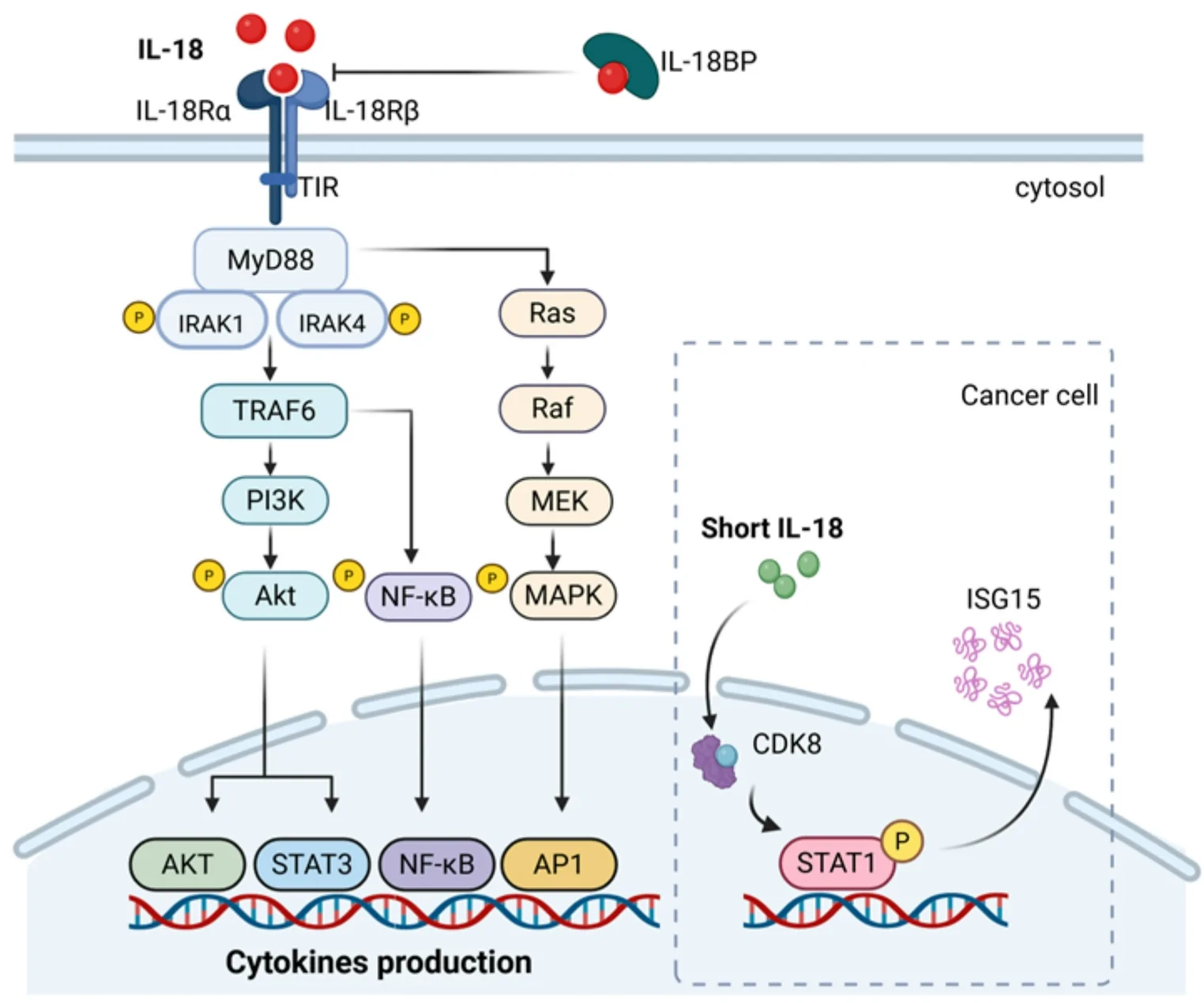
IL-18 signal transduction. IL-18 forms a signaling complex by binding to its heterodimeric receptor complex, composed of IL-18Rα and IL-18Rβ. This engagement recruits the adaptor protein MyD88 via TIR domain interactions, leading to the sequential activation of downstream signaling molecules, including IRAK1, IRAK4, and TRAF6. The signal then diverges to activate pivotal downstream pathways such as the NF-κB, MAPK, and PI3K cascades, ultimately driving the expression of pro-inflammatory cytokines and other cellular responses. A critical regulatory mechanism is represented by IL-18BP, a soluble high-affinity decoy receptor that neutralizes IL-18 activity, thus acting as a primary endogenous inhibitor. Additionally, the figure highlights a distinct mechanism relevant to cancer biology, involving a short isoform of IL-18 and its interplay with molecules like ISG15 and CDK8. ISG15, Interferon-stimulated gene 15; CDK8, Cyclin-dependent kinase 8

Beyond the canonical MyD88-dependent NF-κB and MAPK pathways, IL-18 can engage several non-canonical pathways in a cell-type- and context-specific manner. These include activation of tyrosine kinase 2 (Tyk2) and subsequent signal transducer and activator of transcription (STAT) signaling, direct STAT3 phosphorylation, nuclear translocation of nuclear factor of activated T cells 4 (NFATc4), and activation of the phosphatidylinositol-3-kinase (PI3K)-Akt-mechanistic target of rapamycin (mTOR) axis, which promotes cell survival, proliferation, and enhanced effector functions [11]. More recently, a distinct 15-kDa isoform of mature IL-18 has been identified that induces STAT1 phosphorylation in specific tumor microenvironments, thereby potentiating NK cell mobilization and antitumor immunity [22]. Thus, the multifaceted nature of the IL-18 signaling cascade, encompassing both canonical and non-canonical mechanisms, provides the foundation for its profound impact on augmenting both host-defensive and pathological immune responses across various physiological and disease contexts (Fig. 2).

### Balance of IL-18 and IL-18BP

The discovery of IL-18BP in 1999 revealed a unique negative-feedback loop within the IL-1/IL-18 cytokine family [26]. The biological activity of IL-18 is tightly controlled by IL-18BP, a constitutively secreted soluble decoy receptor. IL-18BP binds mature IL-18 with extraordinary picomolar affinity, which is approximately 20–50-fold higher than the affinity of IL-18 for its signaling receptor chain IL-18Rα [27]. This kinetic superiority, coupled with an extremely slow dissociation rate, enables IL-18BP to function as a potent scavenger, irreversibly sequestering free IL-18 and blocking its interaction with the cell-surface IL-18Rα/β heterodimer. In healthy individuals, circulating IL-18BP concentrations (typically 1–20 ng/mL) substantially exceed those of bioactive free IL-18 (< 100 pg/mL) [28], ensuring that the majority of IL-18 is maintained in an inactive, bound state and preventing unchecked systemic proinflammatory activity. This high-affinity, molar-excess mechanism serves as a vital endogenous safeguard against excessive IL-18-mediated inflammation.

Clinically, an increased ratio of free IL-18 to IL-18BP, which reflects either overproduction of IL-18 or insufficient IL-18BP, has emerged as a hallmark of numerous autoimmune and inflammatory diseases, establishing the IL-18/IL-18BP axis as both a sensitive disease-activity biomarker and a promising therapeutic target [29, 30]. Recombinant human IL-18BP (tadekinig alfa) has advanced into clinical trials, demonstrating the translational potential of amplifying this endogenous regulatory mechanism to suppress dysregulated IL-18 signaling [31].

### IL-18 in Innate and Adaptive Immune Regulation

**Fig. 3:**
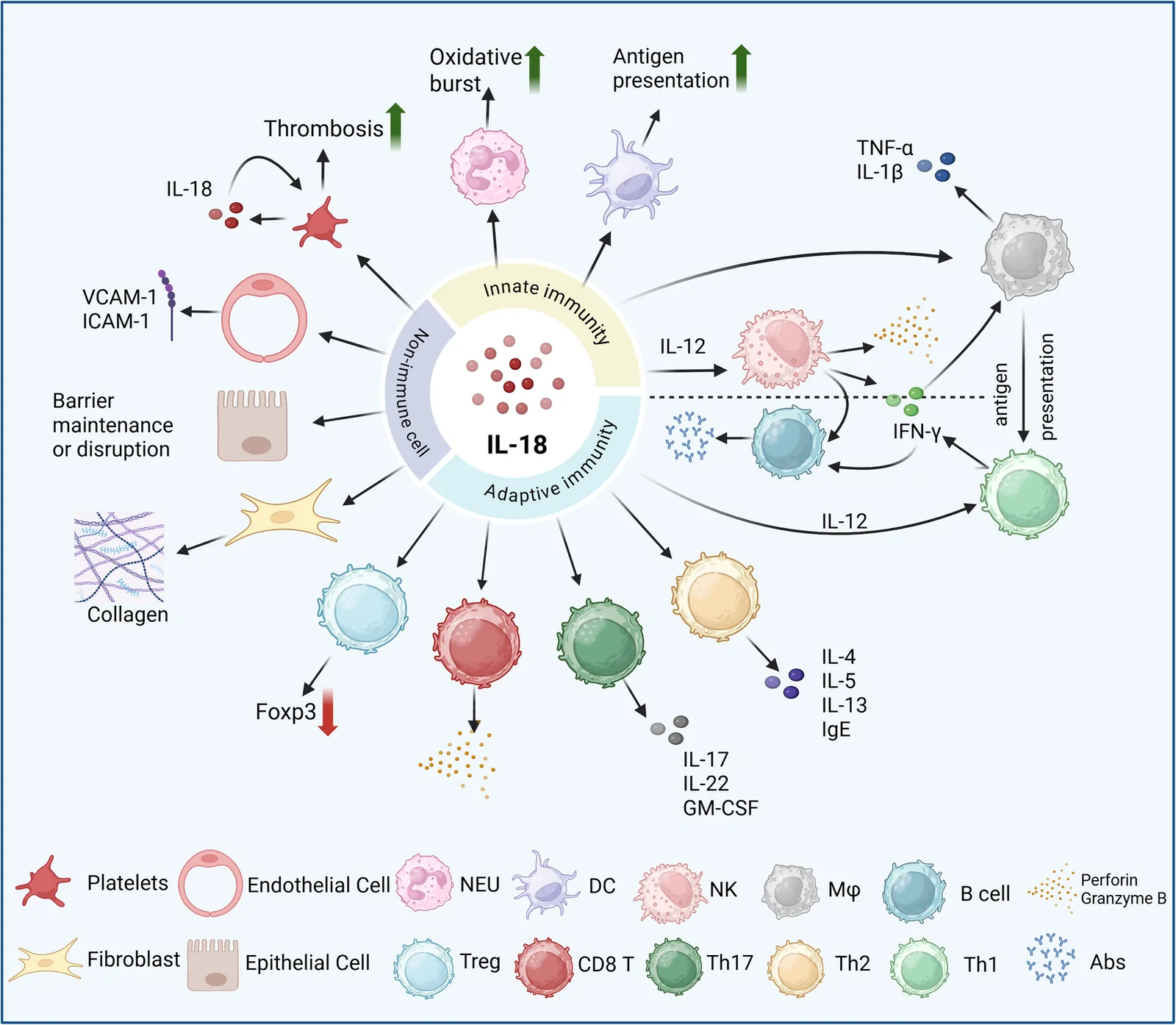
IL-18 as a central regulator of innate and adaptive immune responses. IL-18 exerts broad immunostimulatory effects on both innate and adaptive immune cells. It activates neutrophils, macrophages, dendritic cells, and NK cells, and, in synergy with IL-12, potently induces IFN-γ production by NK cells and Th1 cells. Beyond its canonical Th1-polarizing role, IL-18 displays pleiotropic activities by directly or indirectly supporting Th2 and Th17 differentiation in a context-dependent manner. In allergic settings, IL-18 promotes IL-4 and IL-13 production, whereas in autoimmune settings it enhances IL-17, IL-22, and GM-CSF production by Th17 cells. Additionally, IL-18 amplifies CD8⁺ T cell cytotoxic activity, boosts antibody production by B cells, and inhibits Treg cell function via suppression of Foxp3 expression. In non-hematopoietic cells, IL-18 upregulates adhesion molecules, such as VCAM-1 and ICAM-1, on endothelial cells, compromises epithelial barrier integrity, and promotes collagen synthesis by fibroblasts, thereby contributing to tissue inflammation and remodeling. GM-CSF, Granulocyte-Macrophage Colony-Stimulating Factor; Foxp3, Forkhead Box P3; VCAM-1, Vascular Cell Adhesion Molecule 1; ICAM-1, Intercellular Adhesion Molecule 1

IL-18 plays a pivotal role in bridging innate and adaptive immunity and exerts diverse effects on both immune and non-immune cell types, contributing to host defense, inflammation, and tissue remodeling (Fig. 3).

#### Innate and Adaptive Immunity

IL-18 exerts powerful immunostimulatory effects on multiple innate immune populations. It activates neutrophils by enhancing their chemotaxis, degranulation, and oxidative burst [32]. In macrophages and dendritic cells, IL-18 promotes maturation, antigen presentation, and stimulates the release of additional pro-inflammatory mediators, thereby establishing essential defenses against intracellular pathogens such as viruses and certain bacteria [33]. NK cells are especially sensitive to IL-18, which promotes their proliferation, cytotoxic activity, and IFN-γ production [34].

In the adaptive immune system, IL-18 directly promotes Th1 cell differentiation in synergy with IL-12, thereby augmenting the cytotoxic clearance of infected or malignant cells [35]. IL-18 also enhances the cytotoxic activity and survival of CD8^+^ cytotoxic T lymphocytes (CTLs), thereby reinforcing cell-mediated immunity [36]. Although traditionally linked to Th1 responses, IL-18 exhibits context-dependent functional plasticity. In the absence of IL-12 or in specific microenvironments, IL-18 can instead support Th2 responses by promoting IL-4, IL-5, and IL-13 production along with IgE synthesis [37] or drive Th17, especially pathogenic Th17 (pTh17) differentiation, contributing to autoimmunity and chronic inflammation [38]. B cells, which exert diverse roles in tissue homeostasis and autoimmune disease pathogenesis [39, 40], are also regulated by IL-18. In the presence of IL-12 or T cell help, IL-18 enhances antibody production, including class-switched isotypes such as IgG2a in mice [41]. Notably, IL-18 suppresses regulatory T (Treg) cell differentiation and function, possibly by inhibiting transforming growth factor-β (TGF-β)-induced Foxp3 expression, which reduces immune tolerance and amplifies inflammatory responses [42].

#### Effects on Non-Immune Cells

Beyond its influence on diverse immune cell populations, IL-18 can act directly on structural and barrier cells to modulate tissue inflammation and repair. In endothelial cells, IL-18 upregulates the expression of adhesion molecules such as VCAM-1 and ICAM-1, thereby promoting leukocyte adhesion and extravasation into inflamed tissues [43]. IL-18 compromises epithelial barrier function in the intestine and lungs by disrupting tight junction proteins, including claudins and occluding, a pathogenic process implicated in in IBD [44] and asthma [45]. In fibroblasts, IL-18 drives proliferation and extracellular matrix deposition, particularly type I and III collagen, thereby fostering fibrosis in chronic inflammatory settings such as RA, systemic sclerosis (SSc), and atherosclerotic plaques [4, 46].

A more recent study has revealed that, in platelets, IL-18 functions as a potent inducer of activation and thrombosis by binding to the surface receptor IL-18Rα. This interaction triggers intracellular signaling cascades that amplify critical processes, including aggregation, granule secretion, and integrin activation. Additionally, activated platelets release pre-stored IL-18, generating a pathogenic positive feedback loop that sustains platelet hyperreactivity. This amplification is particularly prominent in patients with coronary artery disease (CAD), a population that typically display elevated circulating IL-18 levels [47].

### IL-18 in the Metabolic Regulation of Immune Cells

Beyond its well-characterized role in regulating innate and adaptive immune responses via canonical proinflammatory signaling pathways, a growing and dynamic body of research has revealed an additional function of IL-18 to directly modulate cellular metabolism in immune cells, thus modulating their activation, differentiation, survival, and effector functions. These metabolic effects are tightly coupled to the bioenergetic and biosynthetic requirements of IL-18-driven immune activation, highlighting a bidirectional interplay between cytokine signaling and immunometabolism. This provides a compelling framework for understanding how IL-18 sustains chronic inflammation in autoimmune diseases and opens new avenues for therapeutic intervention through metabolic targeting.

Recent studies show that IL-18 drives macrophage polarization toward a pro-inflammatory M1-like phenotype [48]. This process is tightly coupled with metabolic reprogramming, marked by enhanced glycolysis and reduced oxidative phosphorylation (OXPHOS). The sustained glycolytic state is maintained through upregulation of hypoxia-inducible factor 1-alpha (HIF-1α), which promotes high glycolytic flux [49].

Short-term exposure to IL-18 triggers a robust metabolic enhancement in NK cells, characterized by simultaneous increases in glycolytic flux and OXPHOS. This dual metabolic reprogramming is orchestrated by the mTORC1-SREBP signaling, leading to increased de novo lipid synthesis and upregulated surface expression of essential nutrient transporters (e.g., GLUT1, CD71, CD98). IL-18-primed NK cells exhibit mitochondrial biogenesis and greater spare respiratory capacity. These metabolic adaptations are indispensable for maintaining cytotoxicity and IFN-γ secretion, particularly within nutrient-poor tumor microenvironments or during chronic viral infections, while inhibition of either glycolysis or OXPHOS markedly diminishes IL-18-enhanced NK cell effector functions [50].

IL-18 also exerts diverse effects on T cell metabolism and function, promoting enhanced bioenergetics that support superior effector and memory responses. These immunomodulatory actions are highly lineage-dependent. In Th1 cells, IL-18 cooperates with IL-12 to potentiate maximal IFN-γ production, a process dependent on mTORC1 signaling and a shift toward aerobic glycolysis [4, 51, 52]. Our recent work shows that IL-18 potentiates pTh17 cell differentiation through Bhlhe40-mediated regulation of glycolytic influx [53]. Additionally, IL-18 opposes Treg cell development by suppressing Foxp3 expression and inducing a phenotypic switch. It redirects Tregs from their typical dependence on oxidative phosphorylation toward glycolysis, thereby converting them into pro-inflammatory effector-like cells and abrogating their suppressive function [42, 54].

In summary, IL-18 acts as a central immunometabolic regulator, shifting both innate and adaptive immune cells toward a high-energy glycolytic state with increased respiratory capacity to enable rapid and robust effector functions. Simultaneously, it suppresses oxidative, anti-inflammatory programs in regulatory populations. This metabolic reprogramming underlies the dual role of IL-18 in promoting protective immunity while also contributing to the pathogenesis of chronic inflammatory and metabolic diseases.

## Roles of IL-18 in Inflammation and Autoimmune Pathogenesis

### IL-18 in Immune Dysregulation

Substantial evidence implicates IL-18 as a key proinflammatory cytokine and pathogenic driver across a wide range of autoimmune and inflammatory diseases. Markedly elevated levels of total and bioactive free IL-18 have been consistently detected in serum, synovial fluid, cerebrospinal fluid, and affected tissues of patients with RA, SLE, AOSD, MAS, IBD, multiple sclerosis (MS), psoriasis, SS, and related conditions [55–61]. In MAS complicating systemic juvenile idiopathic arthritis (sJIA), AOSD, or SLE, circulating free IL-18 levels often exceed 10,000–100,000 pg/mL, far surpassing those of other cytokines and serving as one of the most specific diagnostic and prognostic biomarkers in these conditions [10, 62, 63].

**Fig. 4:**
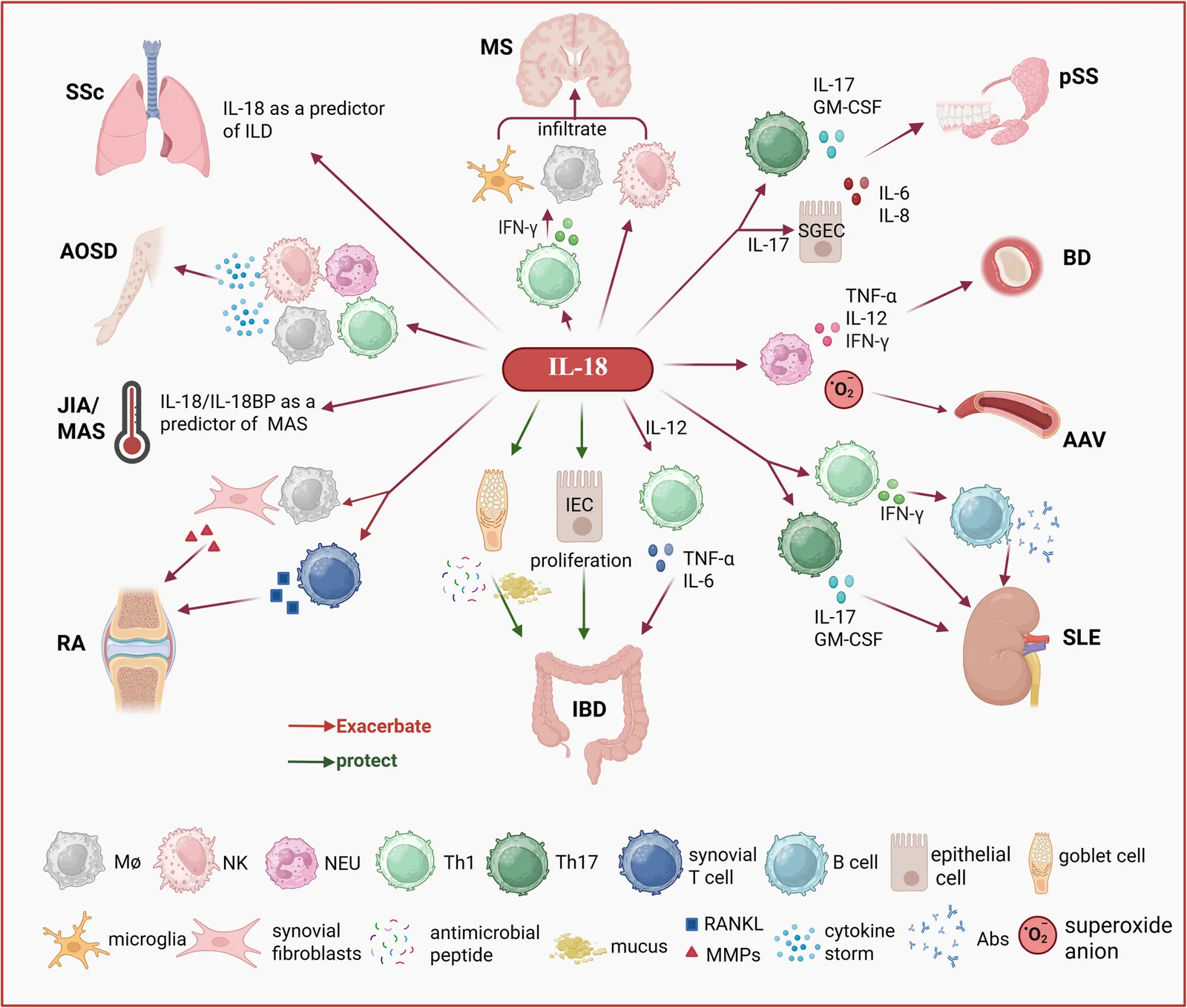
The pleiotropic roles of IL-18 in inflammation and autoimmune diseases. IL-18 predominantly functions as a potent pro-inflammatory cytokine that drives the differentiation and activation of diverse immune effector subsets, including Th1, Th17, NK cells, macrophages, and neutrophils, thereby amplifying the production of downstream inflammatory mediators and contributing to tissue damage in a wide range of autoimmune and autoinflammatory diseases. In MS, IL-18 facilitates the infiltration of NK cells and macrophages into the CNS. In SS, it promotes Th17-mediated epithelial inflammation through enhanced IL-17 and GM-CSF production. In BD and AAV, it fuels neutrophil-driven vascular inflammation. In SLE, it enhances Th1-dependent B-cell activation and autoantibody production, exacerbating the renal pathology. In RA, it stimulates synovial T cells and macrophages to secrete MMPs and RANKL, thereby accelerating joint destruction. In SSc, elevated IL-18 levels predict ILD. In AOSD and MAS, it acts as a central driver of life-threatening cytokine storms, with the free IL-18/IL-18BP ratio as a strong predictor of MAS in patients with JIA. In contrast, IL-18 exerts protective effects in IBD under homeostatic or certain inflammatory conditions by promoting IEC proliferation, enhancing goblet-cell mucus production, and inducing antimicrobial peptide secretion, thereby reinforcing mucosal barrier integrity. However, this role is highly context-dependent, as IL-18 can paradoxically exacerbate IBD by driving pathogenic Th1 responses in synergy with IL-12, highlighting its dual function in immunity and tissue homeostasis. Red arrows denote pathways by which IL-18 exacerbates inflammation or drives pathogenic immune responses, whereas green arrows indicate protective or homeostatic functions of IL-18

Through multiple mechanisms, IL-18 sustains systemic and organ-specific inflammation as well as perpetuates inflammatory loops and tissue damage in various autoimmune diseases. In SLE and SS, IL-18 facilitates Th1 and pTh17 differentiation, promoting B cell activation, autoantibody production, immune complex deposition, and tissue injury [53, 64]. Similarly, in RA, BD, and AOSD, IL-18 intensifies the inflammatory milieu by orchestrating Th1 responses, neutrophil activation, and excessive IFN-γ secretion [55, 65, 66]. In MAS, IL-18 potentiates NK cell dysfunction, cytotoxic lymphocyte activity, endothelial activation, and synergy with IFN-γ to fuel uncontrolled macrophage activation and hemophagocytosis [67]. In IBD, excess IL-18 promotes Th1/Th17 polarization, amplifies IFN-γ and TNF-α from CD4⁺ T cells and innate lymphoid cells, and sustains epithelial barrier breakdown [68]. For AAV, IL-18 perpetuates neutrophil-mediated vascular inflammation and tissue damage, while in MS, it acts as a critical upstream amplifier of innate and adaptive immune responses (Fig. 4).

Beyond chronic autoimmunity, IL-18 plays a critical role in acute hyperinflammatory syndromes and cytokine storms, including severe viral infections (e.g., COVID-19 and influenza), NK/T-cell lymphomas, and cytokine release syndrome or neurotoxicity associated with CAR-T cell or bispecific antibody therapies [69–71]. Recent studies highlight the ratio of free IL-18 to IL-18BP as a superior biomarker for monitoring disease activity, flare prediction, and therapeutic response monitoring in RA, sJIA/AOSD, IBD, and related disorders [72]. Thus, dysregulated IL-18 signaling occupies a central position at the intersection of chronic autoimmunity, episodic hyperinflammation, and life-threatening cytokine storms, establishing the IL-18/IL-18BP axis as a potential biomarker for disease activity and a feasible therapeutic target.

### IL-18 in Various Autoimmune Diseases

#### Systemic Lupus Erythematosus (SLE)

IL-18 exerts a profound and pleiotropic influence on the immunopathogenesis and clinical progression of SLE. Compelling clinical data reveal a robust association between augmented serum and urinary IL-18 concentrations and heightened disease activity in SLE patients, with particularly pronounced elevations observed in those afflicted with lupus nephritis (LN) and neuropsychiatric SLE (NPSLE) [73, 74]. Systematic reviews and meta-analyses affirm that circulating IL-18 is significantly higher in SLE cohorts than in healthy controls, with the most pronounced elevations observed in active disease and renal involvement, thereby establishing IL-18 as a sensitive biomarker of flare risk and organ damage [75]. In the context of NPSLE, elevated free IL-18 in cerebrospinal fluid has been linked to seizure disorders, implicating IL-18 in compromising blood-brain barrier integrity and fomenting neuroinflammatory cascades, hallmarks of this neurological complication [74]. Genetic association studies further strengthen the causal framework, revealing that functional single-nucleotide polymorphisms in the IL18 and IL18R1 loci confer increased SLE susceptibility, modulate transcriptional activity, and correlate with heightened disease severity and autoantibody titers [76, 77]. Within the renal milieu of LN, overexpression of IL-18 by tubular epithelial cells precipitates macrophage activation and the upregulation of profibrotic factors, including TGF-β and collagen, thereby intensifying glomerular inflammation, mesangial proliferation, and interstitial fibrosis [78, 79].

Preclinical validation is provided by lupus-prone MRL/MpJ-Fas^lpr^ mice, which exhibit lymphocyte hyperresponsiveness to IL-18, accelerated lymphadenopathy, and exacerbated glomerulonephritis [80]. Genetic or pharmacological blockade of the IL-18Rα in these models significantly delays disease onset, attenuates proteinuria, reduces glomerular immune-complex deposition, and improves survival [76]. Building on this, Bossù et al. demonstrated that DNA vaccination with a plasmid encoding murine pro-IL-18 (pEF2-IL-18) induces high-titer neutralizing autoantibodies, effectively suppressing IFN-γ levels, reducing lymphoproliferation, attenuating renal damage, and prolonging survival in MRL/lpr mice [81]. These findings collectively establish IL-18 as a key mediator of SLE progression, reinforcing the rationale for targeting IL-18 as a promising therapeutic intervention. Taken together, these clinical, genetic, and experimental observations establish IL-18 as a pivotal orchestrator in SLE, providing a compelling rationale for therapeutic strategies targeting IL-18.

#### Rheumatoid Arthritis (RA)

Patients with RA exhibit significantly elevated levels of IL-18 in their serum, synovial fluid, and synovial tissues, which are substantially higher than those found in healthy individuals or patients with osteoarthritis. This overexpression of IL-18, combined with elevated levels of IL-18R and IL-18BP, creates a self-reinforcing proinflammatory feedback loop in the articular microenvironment, thereby sustaining chronic synovitis and progressive joint destruction [82]. Genetic underpinnings further support the involvement of IL-18 in etiological relevance, as polymorphisms in the IL18 gene locus have been robustly linked to enhanced RA susceptibility and severity, indicating that genetic dysregulation may contributes to its pathological overexpression [83]. Recent investigations have extended these findings, revealing its additional involvement in promoting angiogenesis within RA synovium, thereby facilitating vascular proliferation that sustains nutrient supply to hyperplastic tissues and exacerbates inflammatory infiltration [84].

One key mechanism by which IL-18 exacerbates RA is through its promotion of osteoclast formation and resulting bone destruction. IL-18 stimulates resident macrophages and synovial fibroblasts to produce matrix metalloproteinases (MMPs) and prostaglandins, thereby contributing to the degradation of cartilage and bone in affected joints [85]. In addition, IL-18 promotes the expression of receptor activator of nuclear factor κB ligand (RANKL) by synovial T lymphocytes, which supports osteoclast differentiation and bone-resorbing activity [86, 87]. This destructive cascade is amplified through IL-18-mediated induction of TNF-α and IL-1β in synovial tissues, creating a self-perpetuating circuit that accelerates joint erosion. These findings have been confirmed in animal models. Studies using IL-18Rα knockout mice demonstrated that IL-18Rα deficiency significantly attenuates arthritis severity, diminishes synovial infiltration by CD4⁺ T cells and macrophages, lowers levels of circulating proinflammatory cytokines and suppresses MMP-3 expression [88]. In conclusion, IL-18 contributes to RA pathogenesis through its dual role in promoting inflammation and bone erosion. Future research should explore the interplay between IL-18 and other cytokines, as well as its role in RA subsets with distinct molecular profiles.

#### Behçet’s Disease (BD)

Behçet’s disease (BD), a chronic systemic vasculitis characterized by recurrent mucocutaneous ulceration, uveitis, and multisystem vascular inflammation, is increasingly recognized as an IL-18-driven inflammatory disorder. Numerous clinical studies have documented markedly elevated circulating IL-18 concentrations in active BD, with serum levels often exceeding those of healthy controls by several fold and correlating positively with validated disease activity scores and severe manifestations, including posterior uveitis, retinal vasculitis, neurological involvement, and large-vessel vasculitis [89]. Elevated IL-18 is also detected in bronchoalveolar lavage fluid and lesional skin, underscoring its role in both systemic and organ-specific inflammation [90]. Genetic studies further implicate IL-18 dysregulation in BD susceptibility, with hyperfunctional promoter polymorphisms that increase its transcriptional activity [91]. Consistent with these findings, IL-18 levels increase markedly during disease flares, especially among younger patients (< 40 years) or those with severe presentations, such as genital ulceration, erythema nodosum, or uveitis. C-reactive protein (CRP) also increases reliably during active disease, outperforming erythrocyte sedimentation rate (ESR) as a marker of inflammation. In contrast, IL-8 and macrophage inflammatory protein-1α (MIP-1α) show no consistent correlation with disease activity. These findings support the potential of IL-18 as a biomarker for flare detection and a future therapeutic target, while establishing CRP as a more reliable indicator of inflammation in BD than ESR [92].

A critical non-lymphoid effector pathway involves hyperactivation of neutrophils. IL-18 primes neutrophils for enhanced reactive oxygen species (ROS) generation, degranulation, and NETosis, processes directly implicated in endothelial injury, microthrombosis, and the characteristic occlusive vasculopathy of BD [93]. In pulmonary manifestations, analysis of bronchoalveolar lavage fluid reveals IL-18 mRNA overexpression accompanied by IFN-γ-driven lymphocyte infiltration [90]. While its genetic and molecular links to vascular inflammation are well-characterized, further investigation is needed to elucidate context-dependent pro- and anti-inflammatory effects and to refine IL-18-targeted therapeutic strategies for this multifactorial disorder.

#### Adult-Onset Still’s Disease (AOSD)

Adult-onset Still’s disease (AOSD) is one of the most prominent examples of IL-18-driven systemic hyperinflammation. Patients with active AOSD exhibit dramatically elevated serum levels of free IL-18 compared to those in healthy individuals [94]. These high concentrations correlate strongly with disease activity scores, systemic inflammation markers and ferritin levels [95]. Notably, IL-18 outperforms all conventional inflammatory parameters in distinguishing AOSD from sepsis, even when fever and hyperferritinemia overlap, making it the most specific biomarker currently available for both diagnosis and differential diagnosis of AOSD [96]. The compensatory increase in IL-18BP is inadequate to neutralize the excessive molar surplus of free IL-18, indicating a failure of endogenous regulatory mechanisms that perpetuates uncontrolled inflammation [97].

In pathogenesis, IL-18 acts synergistically with IL-1β and IL-6 to hyperactivate macrophages and neutrophils, driving massive release of TNF-α and IFN-γ, which can trigger a cytokine storm [98]. Histopathological studies of lymph nodes and skin reveal pronounced IL-18 overexpression in paracortical regions, accompanied by characteristic paracortical hyperplasia, T-zone expansion, and vascular proliferation [99]. Emerging evidence further implicates that NLRP3 inflammasome hyperactivation and neutrophil-derived proteinase-3-mediated extracellular cleavage as primary mechanisms generating mature IL-18 in AOSD [100]. Early clinical data with recombinant human IL-18BP and direct IL-18 inhibitors have shown rapid ferritin normalization, resolution of MAS, and steroid-sparing effects [101]. These findings provide compelling proof-of-concept for the central pathogenic role of the IL-18 pathway in this rare disease.

#### Juvenile Idiopathic Arthritis (JIA) and Macrophage Activation Syndrome (MAS)

Juvenile idiopathic arthritis (JIA), particularly its systemic subtype (sJIA), is a severe autoinflammatory disorder, characterized by persistent arthritis, daily spiking fevers, transient salmon-colored rash, serositis, and hepatosplenomegaly. A life-threatening complication of sJIA is MAS, a hyperinflammatory syndrome with high mortality driven by cytopenias, coagulopathy, marked hyperferritinemia, and multi-organ failure [102]. IL-18 has emerged as a critical mediator in sJIA pathogenesis, functioning as both a biomarker for disease severity and a predictor of MAS risk. Patients with active sJIA exhibit dramatically and persistently elevated levels of total and free IL-18 compared to those with other JIA subtypes [103]. These concentrations correlate strongly with systemic inflammation markers, including ferritin, CRP as well as disease activity, making it a valuable prognostic marker [104]. Notably, persistent IL-18 elevation during clinical remission signals ongoing immune dysregulation and heightened risk of disease relapse [105].

In JIA, dysregulated activation of the NLRP3 inflammasome in macrophages and monocytes leads to excessive IL-18 secretion [106]. Patients with JIA often exhibit disproportionately low IL-18BP level relative to IL-18, leading to increased bioavailability of free IL-18 and potentially exacerbating the inflammatory response [107]. This imbalance is linked to a heightened risk of MAS, particularly during disease flares. Future research should aim to elucidate the role of IL-18 in distinct JIA endotypes and the specific triggers of MAS. Genetic studies of IL-18 or NLRP3 polymorphisms may help identify high-risk individuals, while longitudinal monitoring of IL-18 and IL-18BP levels could further improve risk stratification and guide personalized therapy.

#### Anti-Neutrophil Cytoplasmic Antibody (ANCA)-Associated Vasculitis (AAV)

ANCA-associated vasculitis (AAV) is characterized by pauci-immune necrotizing inflammation of small- and medium-sized vessels, with frequent renal and pulmonary involvement. IL-18 has emerged as a key amplifier of neutrophil-driven vascular injury in AAV. Serum IL-18 concentrations are significantly elevated in active AAV compared with healthy controls and disease-remission states, correlating positively with disease activity [108]. In AAV-affected kidneys, IL-18 is highly expressed by tubular epithelial cells, infiltrating macrophages, and cells within glomerular crescents. As a result, urinary IL-18 reflects local intrarenal production and serves as a sensitive, non-invasive biomarker of tubular injury and disease activity [109].

At the mechanistic level, IL-18 exacerbates vascular injury by enhancing neutrophil responsiveness. Hewins et al. demonstrated upregulated IL-18 expression in renal tissues of AAV patients, which primes neutrophils for ANCA-induced activation, thereby intensifying neutrophil degranulation and respiratory burst and leading to endothelial damage and glomerular injury [110]. Pressler et al. further emphasized the pivotal role of IL-18 in linking innate immunity and ANCA-mediated neutrophil activation, suggesting that IL-18-dependent inflammatory pathways can perpetuate a self-reinforcing cycle of inflammation and tissue destruction [111]. Novick et al. reported a compensatory elevation of IL-18BP in granulomatosis with polyangiitis (GPA, an AAV subtype), with levels inversely correlating with disease activity. This implies that insufficient IL-18BP during disease flares may permit unchecked neutrophil activation [112]. Preclinical studies in anti-MPO antibody-induced glomerulonephritis models have demonstrated that IL-18 blockade or genetic deletion markedly attenuates neutrophil infiltration, crescent formation, and renal dysfunction, whereas administration of exogenous IL-18 exacerbates injury [110]. Collectively, these clinical, histopathological, and experimental data establish IL-18 as a pivotal bridge between innate immune activation and ANCA-triggered neutrophil cytotoxicity in AAV.

#### Multiple Sclerosis (MS)

Multiple sclerosis (MS) is a chronic autoimmune demyelinating disease of the central nervous system (CNS) characterized by neuroinflammation, blood-brain barrier disruption, and progressive neurodegeneration [113]. IL-18 has emerged as a central proinflammatory mediator in MS pathogenesis. Serum IL-18 concentrations are significantly elevated in patients with MS compared with healthy controls, with levels correlating positively with clinical disability, relapse frequency, and radiological evidence of disease activity, particularly the presence of gadolinium-enhancing lesions on magnetic resonance imaging [114]. Cerebrospinal fluid studies further reveal increased free IL-18 during active disease phases, underscoring a potential contribution to blood-brain barrier compromise and the recruitment of pathogenic leukocytes into the CNS [115]. Genetic studies further support the involvement of IL-18 in MS susceptibility. Specifically, the − 607 C/A promoter polymorphism in the IL-18 gene is linked to greater MS risk in a Turkish cohort [116]. This variant may augment IL-18 production, thereby sustaining a pro-inflammatory milieu that fuels the autoimmune attack on neural tissues. Additional polymorphisms (−137 G/C) and haplotypes within the IL-18/IL-18R locus similarly correlate with altered IL-18 expression and increased risk of primary progressive MS [117].

IL-18 amplifies neuroinflammatory cascades through in MS through diverse mechanisms. It synergizes with CD40–CD40L co-stimulation to potentiate activation of autoreactive CD4⁺ T-cells, driving robust IFN-γ secretion and subsequent polarization toward encephalitogenic Th1 and Th17 phenotypes [118]. This IFN-γ-rich environment activates resident microglia and infiltrating macrophages, upregulates MHC class II and co-stimulatory molecules, and promotes oligodendrocyte apoptosis along with myelin phagocytosis. Additionally, IL-18 boosts NK cell cytotoxicity and stimulates endothelial cells to express adhesion molecules and chemokines, thereby facilitating leukocyte trafficking across the disrupted blood-brain barrier, and exacerbating CNS infiltration and inflammatory damage. These combined effects significantly aggravate neuroinflammation and contribute to irreversible neuronal damage [119]. In experimental autoimmune encephalomyelitis (EAE), the primary animal model of MS, deficiency in IL-18 or IL-18Rα markedly mitigates clinical severity, reduces CNS inflammatory infiltrates, and preserves myelin integrity, whereas administration of exogenous IL-18 exacerbates disease [120].

#### Inflammatory Bowel Disease (IBD)

Inflammatory bowel disease (IBD), including Crohn’s disease (CD) and ulcerative colitis (UC), exemplifies the complex nature of IL-18, which exerts both pathogenic and homeostatic functions within the intestinal mucosa [121]. Serum and fecal IL-18 levels are significantly elevated in active IBD and correlate with endoscopic severity, histological inflammation, and clinical disease activity scores [122]. In inflamed lesions, IL-18 is abundantly expressed by lamina propria macrophages, dendritic cells, and intestinal epithelial cells (IECs), primarily following NLRP3, NLRC4, or AIM2 inflammasome activation in response to microbial danger signals or endogenous damage-associated molecular patterns [123]. Neutralization of IL-18 or genetic deletion of IL-18/IL-18Rα markedly attenuates weight loss, mucosal ulceration, and inflammatory infiltrates, whereas administration of mature IL-18 exacerbates disease [124, 125]. Conversely, IL-18 plays essential protective roles that extend beyond its pathogenic function in IBD, functioning critically in both intestinal homeostasis and systemic barrier defense. Under steady-state conditions, IEC-derived IL-18 is constitutively expressed and is vital for maintaining gut homeostasis by promoting goblet cell differentiation, reinforcing the mucus layer, stimulating antimicrobial peptide secretion, and supporting IEC proliferation [126, 127]. Beyond the gut, IL-18 critically mediates tissue repair and antimicrobial defense across various barrier sites. In settings such as acute lung injury and skin wound healing, it directly promotes epithelial regeneration and integrity. During bacterial, viral, or fungal infections, IL-18 is pivotal for recruiting innate immune cells, stimulating the production of antimicrobial peptides, and supporting pathogen-specific T cell responses [128–130].

Therefore, although IL-18 blockade is beneficial for controlling hyperinflammatory conditions, it may be harmful in specific contexts. Particular caution is required in patients with active infections, where inhibition could impair essential host defense responses [28]; in individuals with compromised epithelial barrier function, where it might delay tissue repair; and in healthy barrier tissues, such as the intestinal, pulmonary, and cutaneous epithelia, that depend on IL-18 for homeostatic and restorative functions [4], its blockade risks increased pathogen susceptibility, delayed healing, or disruption of microbial homeostasis [24]. This is contrasted with pathological states driven by excessive IL-18, emphasizing the critical need for context-dependent therapeutic strategies.

#### Sjögren’s Syndrome (SS)

In Sjögren’s syndrome (SS), IL-18 plays a pivotal role in driving exocrine gland inflammation and systemic autoimmunity. Immunohistochemical and transcriptomic analyses of minor salivary gland (MSG) biopsies reveal marked IL-18 overexpression in ductal and acinar epithelial cells, as well as infiltrating mononuclear cells, with expression levels strongly correlating with focus scores, formation of ectopic germinal center (GC)-like structures, and overall histopathological severity [131]. Serum IL-18 concentrations are markedly higher in SS patients compared with healthy controls or sicca controls, and correlate positively with European League Against Rheumatism Sjögren’s Syndrome Disease Activity Index (ESSDAI), serum IgG levels, anti-Ro/SSA and anti-La/SSB autoantibody titers, and the risk of progression to non-Hodgkin lymphoma (NHL), particularly mucosa-associated lymphoid tissue (MALT) lymphoma [53].

In the pathology of SS, IL-18 functions through multiple mechanisms to drive glandular inflammation. IL-18 acts synergistically with IL-17, both commonly co-expressed in SG infiltrates, to drive cis-signaling in SG epithelial cells, leading to robust secretion of IL-6, IL-8, and additional proinflammatory mediators that sustain lymphocytic infiltration and tissue destruction [132]. Strong activation of NLRP3, AIM2, and caspase-1 is observed in SGs of SS, indicating that mature IL-18 release is predominantly inflammasome-dependent [133]. In vitro stimulation of human SGEC lines (HSY, AZA3) with calcium ionophores (e.g., A23187/calcimycin) induces dose-dependent IL-18 secretion, recapitulating intracellular Ca²⁺-mediated inflammasome activation observed in vivo [134]. Notably, IL-18 is preferentially localized to ectopic GC-like structures within SGs, where it supports B-cell survival, class-switch recombination, and autoantibody production [135]. Our recent study has shown that IL-18 directly promotes the differentiation and metabolic reprogramming of pTh17 cells via STAT3 phosphorylation and stabilization of the transcription factor Bhlhe40, thereby increasing glycolytic activity and production of proinflammatory cytokines, particularly GM-CSF, and intensifying tissue injury. Importantly, preclinical studies demonstrate that neutralizing IL-18 with a monoclonal antibody effectively suppresses the pTh17 cell response and ameliorates glandular pathology in SS mouse models [53]. Therefore, targeting the IL-18 may offer promising therapeutic strategies for alleviating glandular destruction in SS.

#### Other Autoimmune Diseases

The pathogenic significance of IL-18 extends across a broad spectrum of autoimmune disorders, where it orchestrates inflammatory, fibrotic, and tissue-destructive processes through diverse disease-specific mechanisms. In SSc, particularly SSc-associated interstitial lung disease (ILD), serum and bronchoalveolar lavage fluid IL-18 levels are markedly elevated and independently predict forced vital capacity decline, fibrosis progression, and mortality [136, 137]. Mechanistically, IL-18 drives TGF-β1-dependent myofibroblast differentiation, excessive collagen synthesis, and alveolar epithelial cell apoptosis, thereby sustaining the fibroproliferative environment typical of SSc-ILD. Most importantly, IL-18 combined with Krebs von den Lungen-6 outperforms traditional pulmonary function tests as an early sensitive biomarker for the detection of ILD [138]. Idiopathic inflammatory myopathies, including polymyositis (PM) and dermatomyositis (DM), and anti-synthetase syndrome, are featured by the overexpression of IL-18 in muscles, which drives macrophage and CD8^+^ T cell activation, ultimately leading to myofiber necrosis. Although standard immunosuppressive treatments reduce IL-18 levels and improve muscle strength, cross-talk with IL-17 sustains chronic inflammation and thus leads to treatment-resistant phenotypes [139, 140].

In Hashimoto’s thyroiditis (HT), IL-18 expression in thyroid follicular cells is closely associated with lymphocyte infiltration [141]. Quantitative RT-PCR and immunohistochemical analyses reveal significantly higher IL-18 mRNA and protein levels in thyroid specimens from HT patients compared with non-autoimmune controls, with expression intensity correlating with the extent of lymphocytic infiltration and follicular destruction [142]. Together, these findings in SSc, idiopathic inflammatory myopathies, and autoimmune thyroiditis highlight the proinflammatory and tissue-damaging role of IL-18 in organ-specific and systemic autoimmunity, while emphasizing its potential as a sensitive, disease-relevant biomarker and promising therapeutic target in these intractable conditions.

## Clinical and Translational Insights: Biomarkers and Therapeutic Targeting

### IL-18 as a Diagnostic Biomarker

As previously noted, IL-18 has emerged as a biomarker of considerable significance in autoimmune diseases. Its persistently elevated concentrations across diverse autoimmune conditions underscore its central pathogenic role, with higher expression correlating with disease activity, flare severity, and the progression of inflammatory cascades. Of particular, the ratio of free IL-18 to IL-18BP, has demonstrated promising predictive value for impending hyperinflammatory states, such as MAS [28]. Despite these associations, the diagnostic utility of IL-18 remains constrained by its limited disease specificity. Elevated IL-18 levels are not unique to autoimmune diseases, they are also observed in infections, malignancies, and other inflammatory conditions [4]. Additionally, correlations between IL-18 levels and disease activity are inconsistent across studies, likely due to heterogeneity in patient populations, disease stages, and measurement technique [143, 144]. This heterogeneity impedes the establishment of standardized clinical cutoffs and compromises its reliability as a consistent marker of disease activity.

Beyond specificity issues, the accurate quantification of IL-18 presents significant challenges that directly impact its reliability and potential as a clinical biomarker. Conventional commercial ELISA kits primarily measure total IL-18 (encompassing both free and IL-18BP-bound forms), whereas the biological activity of IL-18 is predominantly determined by its free form. Given that IL-18BP neutralizes IL-18 with high affinity, free IL-18 levels are extremely low or undetectable in healthy individuals and most pathological states, rendering total IL-18 measurements often inadequate for accurately reflecting true signaling strength and disease activity. In contrast, free IL-18 assays, achieved either by simultaneous measurement of total IL-18 and IL-18BP followed by calculation, or through novel specific immunoassays, more directly capture the bioactive fraction and demonstrate stronger correlations with disease activity, and response to IL-18 blockade, thereby offering superior utility for patient stratification, therapeutic monitoring, and prognostic assessment. Despite showing superior predictive value for disease activity, severity, and treatment response compared to total IL-18, the clinical adoption of free IL-18 as a biomarker has been hindered by technical challenges, limiting its utility in patient stratification for targeted therapies. Many available tests cannot adequately differentiate between the precursor and mature forms of IL-18, introducing substantial variability in measured concentrations [145]. Additional complexities, including methodological variability across assays, sample stability issues, and interference from IL-18BP, further complicate accurate quantification [146]. Therefore, prioritizing the measurement of free IL-18 in biomarker research would significantly improve diagnostic precision and help guide personalized treatment strategies.

### Targeting IL-18 in ‌Preclinical Research

Targeting the IL-18 signaling pathway has emerged as a promising therapeutic approach for various autoimmune and inflammatory diseases, supported by compelling preclinical evidence. In experimental models of SLE, including chromatin-induced lupus and MRL/lpr mice, blockade of IL-18 with a monoclonal antibody has demonstrated marked therapeutic benefits. Treatment significantly improved renal function, as evidenced by reduced proteinuria and serum creatinine levels, and substantially lowered autoantibody titers, including anti-nuclear antibodies (ANA) and anti-dsDNA antibodies. Histologically, it attenuated glomerular damage and immune complex deposition in the kidneys [53, 76]. In RA, genetic ablation or pharmacologic inhibition of IL-18Rα dramatically reduces disease severity. In the collagen-induced arthritis model, IL-18Rα knockout mice exhibit lower clinical arthritis scores, diminished cartilage degradation, and reduced subchondral bone erosion. Histologically, these protective effects are accompanied by decreased synovial hyperplasia, reduced infiltration of CD4^+^ T cells and F4/80^+^ macrophages, and lower levels of pro-inflammatory cytokines including IL-6, TNF-α, IFN-γ, and IL-18 both systemically and locally [86, 88]. Clinically, elevated IL-18 concentrations in synovial fluid correlates with resistance to conventional disease-modifying antirheumatic drugs and poorer response to TNF-α inhibitors, suggesting its potential as a biomarker for identifying treatment-refractory RA [147]. In experimental SS, both local (administered via salivary retrograde cannulation) and systemic (intraperitoneal) IL-18 neutralization with a monoclonal antibody yielded substantial efficacy. This intervention led to a notable improvement in SG function, as measured by increased salivary flow rates, while reducing lymphocytic infiltration and tissue destruction histologically. Systemically, it lowered serum autoantibody levels (anti-SSA and anti-M3R) and reduced germinal center B cells and plasma cells [53]. In AAV, IL-18 pathway inhibition markedly suppresses neutrophil priming, degranulation, resulting in reduced endothelial damage and attenuation of necrotizing vasculitis in experimental models [110]. In IBD, the role of IL-18 is context-dependent and compartment-specific. While global IL-18 or IL-18Rα deficiency protects against DSS-induced colitis, mice deficient in IL-18 bp develop severe, spontaneous colitis with goblet cell loss and barrier disruption. This phenotype was completely abrogated in double-mutant mice lacking IEC-specific IL-18Rα (IL-18 bp^−/−^; IL-18Rα^Δ/EC^), where IEC-specific IL-18Rα deletion abolished IL-18 signaling, indicating that unchecked IL-18 signaling in IECs drives mucosal inflammation and barrier dysfunction [148]. These findings position epithelial IL-18R signaling as a critical, non-redundant driver in IBD pathogenesis and an attractive therapeutic target (Table 1).

**Table 1:** Preclinical research on IL-18 pathway modulation strategies for autoimmune diseases

Disease	Model	Intervention	Key Therapeutic Effects	Key Mechanisms/Pathological Improvements	References
Systemic Lupus Erythematosus (SLE)	Chromatin-induced lupus model; MRL/lpr mice	Anti-IL-18 neutralizing mAb (i.p.)	Marked improvement in renal function and disease activity	↓ Proteinuria and serum creatinine ↓ Titers of ANA and anti-dsDNA antibodies ↓ Glomerular injury and immune complex deposition	[53, 76]
Rheumatoid Arthritis (RA)	Collagen-induced arthritis (CIA) model	Genetic ablation of IL-18Rα	Profound reduction in arthritis severity	↓ Clinical arthritis scores, cartilage/bone erosion, synovial hyperplasia ↓ Infiltration of CD4⁺ T cells and macrophages ↓ Pro-inflammatory cytokines (IL-6, TNF-α, IFN-γ, IL-18) ↑ Expression of SOCS3 protein (negative feedback regulator)	[88]
Sjögren’s Syndrome (SS)	Murine Sjögren’s-like model	Local or systemic anti-IL-18 neutralizing mAb (i.p.)	Improved salivary gland function and pathology	↑ Salivary flow rate ↓ Lymphocytic infiltration and tissue damage in glands ↓ Anti-SSA and anti-M3R autoantibodies ↓ Germinal center B cells and plasma cells	[53]
ANCA-Associated Vasculitis (AAV)	Human renal biopsies & ex vivo neutrophil assays	IL-18 signaling inhibition	Attenuated necrotizing vasculitis	↑ IL-18 expression in renal podocytes, tubular epithelium, and infiltrating macrophages ↓ TNFα-independent neutrophil priming, degranulation, and NETosis (via p38 MAPK phosphorylation) ↓ Endothelial damage and vascular inflammation	[110]
Inflammatory Bowel Disease (IBD)	DSS-induced colitis	1. IEC-specific deletion-18Rα (Il18r1 ^ΔIEC^)​ 2. Global deletion of IL-18BP (Il18bp^−/−^)​ 3. IEC-specific rescue in hyper-IL-18 background (Il18bp^−/−^; Il18r1^ΔIEC^)	Protection against chemically induced colitis	IL-18 signaling directly on IECs drives pathology Abolishing epithelial IL-18R signaling restores goblet cells and barrier integrity	[148]

*i.p* Intraperitoneal injection, *DSS* dextran sodium sulfate, *SOCS3* Suppressor of Cytokine Signaling 3, *SSA* Sjögren's-syndrome-related antigen A, *M3R* Muscarinic Acetylcholine Receptor

### Targeting IL-18 in Clinical Trials

Building on compelling preclinical evidence, recent clinical trials have investigated IL-18 pathway inhibition as a potential therapeutic strategy for autoimmune and autoinflammatory diseases, including AOSD, BD, sJIA, and IBD. Early-phase studies have evaluated monoclonal antibodies (such as GSK1070806 and camoteskimab) and recombinant IL-18BP (Tadekinig alfa) in these conditions.

A pivotal Phase II trial (NCT02398435) [149] evaluated tadekinig alfa in 23 patients with AOSD. The study successfully met its primary endpoint, demonstrating an acceptable safety profile over 12-weeks, while secondary endpoints provided early evidence of efficacy, including fever resolution and a ≥ 50% reduction in in CRP levels by week 3. The results, published in 2018, established important proof-of-concept for this mechanism in AOSD and highlighted the need for larger confirmatory trials [143]. However, the path to toward broader clinical validation has faced challenges. Tadekinig alfa subsequently advanced to a Phase III randomized withdrawal trial (NCT03113760) [150] in up to 25 pediatric patients with NLRC4-associated MAS or x-linked inhibitor of apoptosis protein (XIAP) deficiency across sites in the United States, Canada, and Europe. This multicenter, double-blind, placebo-controlled study, amended in 2019 to include an initial open-label lead-in phase for all patients, completed primary data collection by late 2023, with full results anticipated in early 2024. An associated open-label extension (NCT03512314) [151] ongoing until October 2024, continues to demonstrate acceptable long-term safety and potential disease stabilization in this extremely rare population.

In a parallel effort, a small Phase Ib study of the anti-IL-18 antibody camoteskimab in AOSD (NCT04752371) [152] was terminated early in 2021 due to recruitment challenges common in ultra-rare diseases, but it provided valuable pharmacokinetic data without new safety concerns. In BD, a Phase II trial (NCT03522662) [153] involving 12 participants assessed the anti-IL-18 monoclonal antibody GSK1070806. The study reported preliminary safety data over 24 weeks while also monitoring clinical outcomes by measuring current disease activity with the Behcet’s Disease Current Activity Form (BDCAF) and tracking cumulative organ damage with the Vasculitis Damage Index (VDI).

For IBD, a Phase I study of GSK1070806 (NCT01035645) [154] in 78 participants confirmed short-term tolerability over a period of 56 to 300 days. However, as an early-phase trial, it was not powered to assess longitudinal efficacy data. More recently, a Phase IIa trial (NCT03681067) [155] in moderate-to-severe Crohn’s disease observed preliminary endoscopic improvement in a subset of patients with elevated IL-18 levels, but heterogeneous cohorts limited generalizability. As of November 2025, the IL-18 inhibition pipeline remains active. GSK1070806 has shifted focus to atopic dermatitis (ongoing Phase IIb with extension NCT04975438) [156], while Tadekinig alfa is preparing regulatory submission for NLRC4/XIAP indications following Phase III readout, and camoteskimab is advancing to Phase IIb in atopic dermatitis (NCT06436183 follow-on) [157] with IBD exploration planned for 2026. Furthermore, an ongoing prospective, single-center observational study (NCT06718049) [158] is evaluating IL-18 and IL-18BP as potential biomarkers for distinguishing sJIA from recurrent autoinflammatory fevers in pediatric patients. This Italian study, which is scheduled to begin in 2025, has an estimated enrollment of 9 participants. Collectively, these Phase I and II trials support the therapeutic rationale for IL-18 pathway inhibition in autoimmune and autoinflammatory diseases. However, they are constrained by small sample sizes, variable endpoints, and, in some cases, incomplete or preliminary efficacy data. Therefore, larger, well-powered, longer-term studies are warranted to fully define the clinical benefit and safety profile of IL-18-targeted therapies (Table 2).

**Table 2:** Clinical trials on IL-18 pathway modulation strategies for autoimmune diseases

NCT Number	Study Title	Condition	Intervention	Phase	Status	Enrollment	Outcome	Country
NCT02398435 [149]	Therapeutic Use of Tadekinig Alfa in Adult-onset Still’s Disease	AOSD	Tadekinig alfa (IL-18BP)	II	Completed	23	Temperature/CRP/Safety at 12 weeks	France, Germany, Switzerland
NCT04752371 [152]	A Study to Evaluate Camoteskimab in Participants with Still’s Disease	AOSD	Camoteskimab (anti-IL-18 mAb)	I	Terminated	5	Temperature/CRP/Safety at 24 weeks	USA, Belgium, Poland, Ukraine
NCT03522662 [153]	Anti IL-18 (GSK1070806) in Behcet’s Disease	BD	GSK1070806 (anti-IL-18 mAb)	II	Completed	12	BDCAF/VDI/ Safety at 24 weeks	United Kingdom
NCT01035645 [154]	First Time in Human Study of Intravenous Interleukin-18 Antibody	IBD	GSK1070806 (anti-IL-18 mAb)	I	Completed	78	Serum IL-18/Safety up to 56–300 days	United Kingdom
NCT03681067 [155]	A Clinical Trial of Antibody GSK1070806 in the Treatment of Patients with Moderate to Severe Crohn’s Disease	CD	GSK1070806 (anti-IL-18 mAb)	I/II	Completed	5	CDAI score/Safety at 24 weeks	United Kingdom
NCT06718049 [158]	Role of IL-18 and IL-18BP in Diagnostic Work-up of sJIA and Autoinflammatory Diseases	sJIA	None (observational)	N/A	Not Yet Recruiting	9	Serum IL-18/IL-18BP levels at 12 months	Italy

The clinical translation of IL-18 blockade faces several critical hurdles that warrant careful consideration. A primary challenge is the need for precise patient stratification to identify individuals most likely to benefit from therapy. Elevated levels of free IL-18, rather than total IL-18, have emerged as a key biomarker in certain hyperinflammatory conditions, such as AOSD, sJIA and MAS. In these contexts, high circulating free IL-18 reflects insufficient neutralization by endogenous regulators and correlates with disease severity [159]. However, in many chronic inflammatory states and malignancies, total IL-18 may be elevated while free IL-18 remains low due to robust sequestration by IL-18BP [27]. This heterogeneity underscores the necessity of measuring free IL-18 levels or the IL-18/IL-18BP ratio as a companion diagnostic to select responsive patient subsets and avoid treating those with already adequately suppressed IL-18 signaling.

These challenges are further complicated by cytokine redundancy and concerns about long-term safety. IL-18 shares overlapping functions with related cytokines, notably IL-12 in driving IFN-γ production and Th1 responses, and IL-1 family members in promoting broader inflammation. Such redundancy may limit the efficacy of IL-18 monotherapy, as compensatory pathways could sustain pathogenic inflammation. This strongly supports the development of biomarker-guided trials that can stratify patients based on their dominant cytokine signature, thereby identifying those most likely to benefit from targeted IL-18 inhibition versus those who may require combination therapy [24]. Moreover, endogenous IL-18BP creates a major barrier because its upregulation in response to inflammation or treatment can rapidly neutralize therapies, a limitation evidenced in early trials of recombinant IL-18 and its direct inhibitors. Novel strategies, including decoy-resistant IL-18 variants and anti-IL-18BP antibodies, are being explored to overcome this limitation [160]. Finally, given that IL-18 contributes to both innate and adaptive antimicrobial immunity, its chronic blockade raises theoretical safety concerns related to impaired host defense against intracellular pathogens and certain viruses [161]. Encouragingly, preclinical models and limited clinical experience suggest that IL-18 inhibition may carry a lower risk of opportunistic infections compared to broad IL-1 blockade, but extended follow-up in diverse patient populations will be required to fully define the safety profile and optimal therapeutic window [162].

## Conclusions and Future Perspectives

Substantial advances have been achieved in understanding the pathogenic contributions of IL-18 to autoinflammatory and autoimmune diseases. However, important knowledge gaps remain that must be addressed to successfully translate these findings into safe and efficacious IL-18-targeted therapies. Key research priorities include clarifying the context-dependent functions of IL-18, particularly its dual pro-inflammatory and tissue-protective effects, such as those observed in intestinal homeostasis. Cutting-edge approaches, including single-cell multi-omics and longitudinal patient cohorts, will be crucial for identifying the molecular determinants that dictate whether IL-18 promotes pathology or confers protection. Furthermore, refining the clinical utility of IL-18 and IL-18BP as biomarkers is essential. This requires the development of standardized assays that reliably distinguish free from bound IL-18 and immature from mature forms to enable accurate disease monitoring. Large-scale, multicenter validation studies are needed to establish normal ranges, define clinically meaningful thresholds, and validate the prognostic performance of these markers, especially in high-risk conditions like MAS, AOSD, and SLE. To improve diagnostic and prognostic specificity, IL-18 assessment should be combined with complementary biomarkers, broader cytokine profiling, or analyses of post-translational modifications. Such integrative strategies will facilitate the development of composite biomarker signatures that more precisely delineate disease subtypes and activity states.

While IL-18-targeted therapies show therapeutic promise for autoimmune and inflammatory conditions, they carry potential risks that warrant caution. Inhibition of IL-18 can increase susceptibility to infections by impairing antimicrobial defense and IFN-γ-driven responses against intracellular pathogens like viruses and bacteria, as evidenced by preclinical models and patients with IL-18 pathway deficiencies [3]. It may also compromise tumor surveillance, potentially allowing cancer progression due to reduced NK cell and CD8^+^ T cell activity, with studies linking IL-18 deficiency to higher tumor incidence in models of colorectal cancer and melanoma [163]. Additionally, paradoxical inflammation could arise from compensatory overactivation of pathways like IL-1β or IL-6, leading to exacerbation of inflammatory pathways observed in some animal models of arthritis and IBD, highlighting the context-dependent nature of IL-18 blockade [164].

Despite their therapeutic potential, IL-18 inhibitors have not achieved widespread clinical adoption compared to IL-1β blockers (e.g., anakinra) and IL-6 inhibitors (e.g., tocilizumab), which are well-established for diseases like RA and cytokine release syndromes. The development of IL-18-targeted therapies has been hindered by the cytokine’s complex biology, which complicates therapeutic window definition and patient stratification; mixed clinical results beyond orphan indications; and safety concerns from IL-18’s protective roles, including increased infection risk and reduced tumor surveillance. Additional barriers include regulatory hurdles, smaller market niches, and limited large-scale trial success, stand in contrast to the rapid advancements, extensive studies, and broad approvals that advanced IL-1β and IL-6 inhibitors.

Future studies should aim for adequately powered, well-stratified trials with longer follow-up to definitively assess efficacy, durability, and long-term safety. Exploration of rational combination strategies (IL-18 plus IL-1β blockade, or IL-18 inhibition plus JAK inhibitors) in patients refractory to current therapies is a high priority. Meanwhile, preclinical studies must continue to optimize IL-18-targeted interventions, carefully evaluate associated risks such as infection susceptibility and impaired tissue repair, and refine predictive biomarkers to identify patients most likely to benefit. The prospective integration of predictive and pharmacodynamic biomarkers to support precision medicine and enrich trial cohorts for likely responders will be pivotal. Collectively, these multifaceted strategies are vital to realizing the full therapeutic potential of IL-18 modulation in autoimmune and inflammatory diseases.

## Data Availability

No new data were generated or analyzed in support of this research.
